# Supplementary figures and images for: Depletion of extracellular asparagine impairs self-reactive T cells and ameliorates autoimmunity in a murine model of multiple sclerosis
Source: eLife. 2026 Jul 8;14:RP107745. doi: 10.7554/eLife.107745 (PMC13345630; doi:10.7554/eLife.107745)

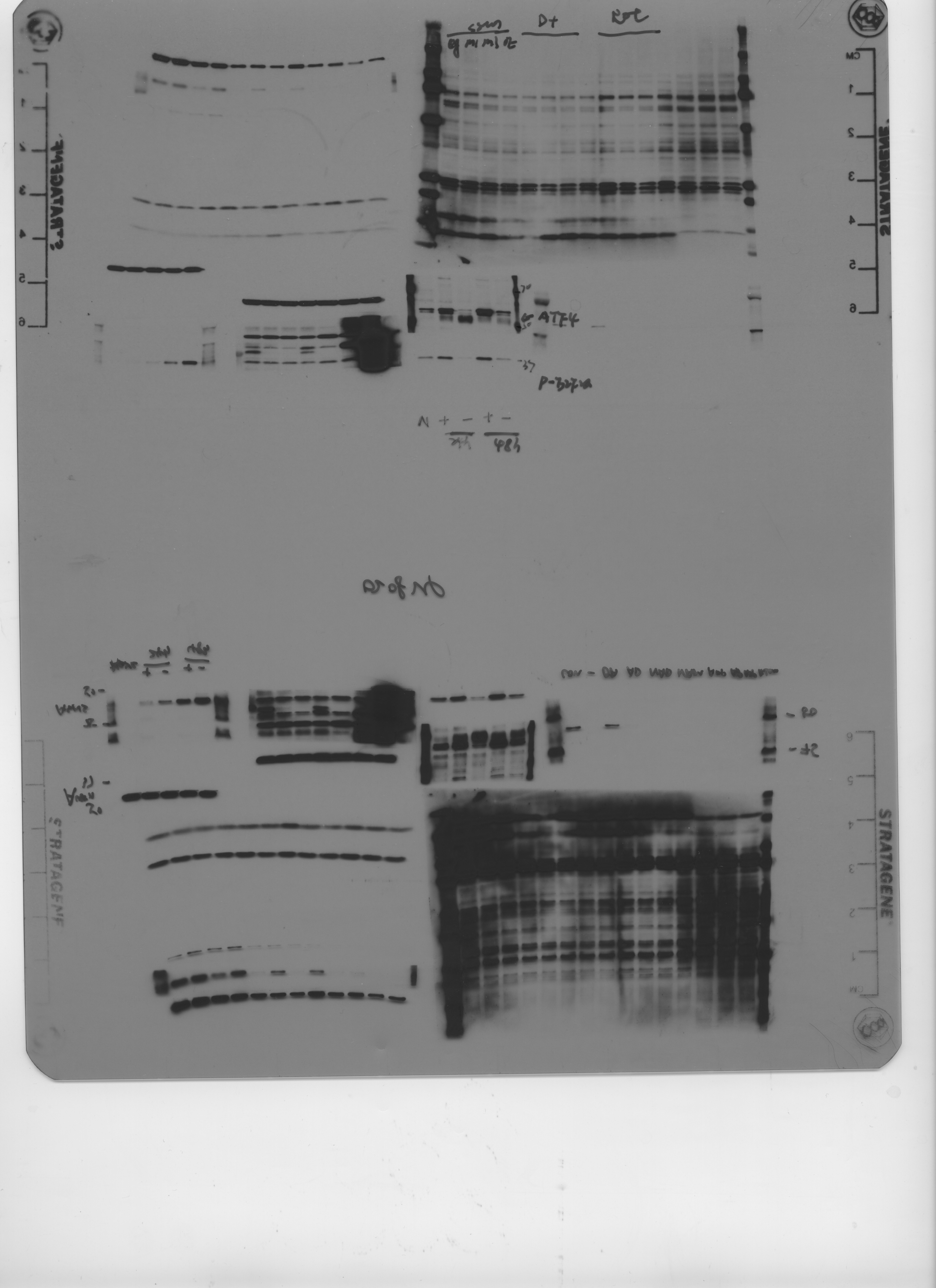

Supplement: Figure 2—source data 2. [file elife-107745-fig2-data2.tif]

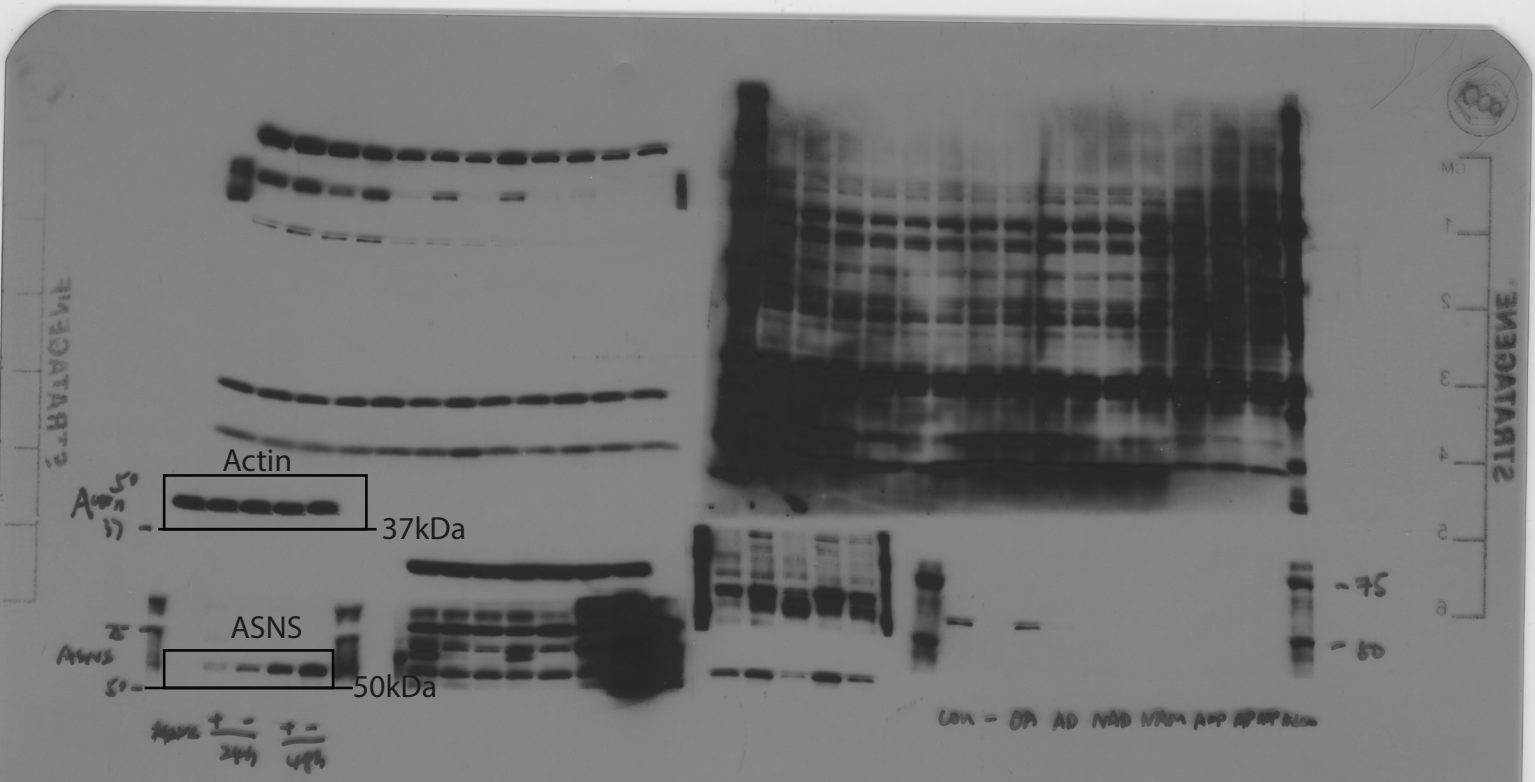

02 of 14

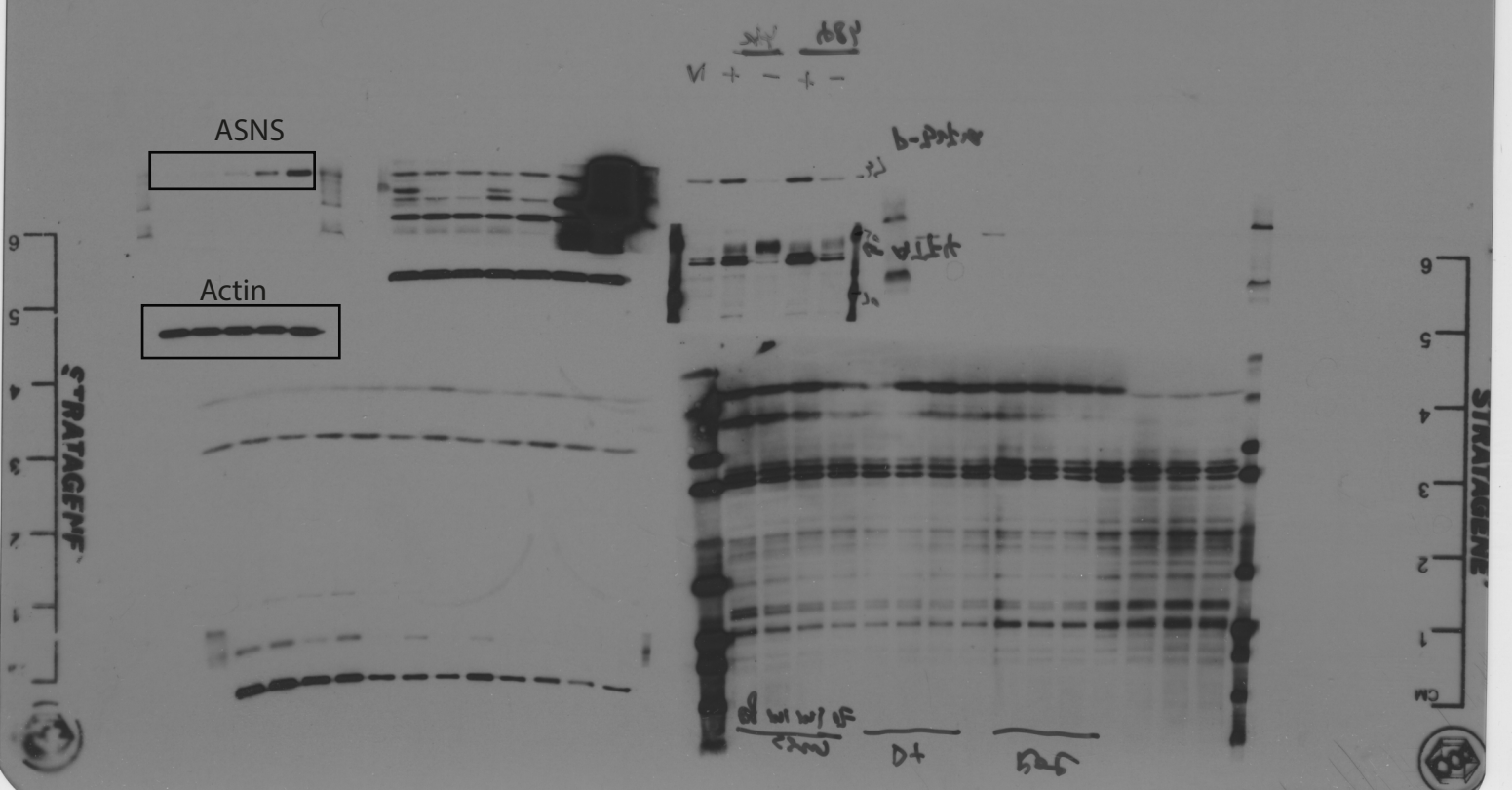

Supplement: Figure 2—source data 3. [file elife-107745-fig2-data3.pdf]
