# Supplementary material for: Depletion of extracellular asparagine impairs self-reactive T cells and ameliorates autoimmunity in a murine model of multiple sclerosis
Source: eLife. 2026 Jul 8;14:RP107745. doi: 10.7554/eLife.107745 (PMC13345630; doi:10.7554/eLife.107745)
Supplement: Peer review history

# Editor report: eLife Assessment

**DOI:** 10.7554/eLife.107745.3.sa0

**Authors:** Tomohiro Kurosaki

**Affiliations:** The University of Osaka Japan

**Keywords:** Convincing, Important

Non-essential amino acids such as glutamine have been known to be required for T cell general activation through sustaining basic biosynthetic processes, including nucleotide biosynthesis, ATP generation, and protein synthesis. In this **important** study, the authors found that extracellular asparagine (Asn) is required not only for T cells to generally refuel metabolic reprogramming, but to produce helper T cell lineage-specific cytokine, for instance, IL17. In particular, the importance of Asn in IL17 production was **convincingly** demonstrated in the mouse experimental autoimmune encephalomyelitis (EAE) model, mimicking human multiple sclerosis disease.

# Referee report: Reviewer #1 (Public review)

**DOI:** 10.7554/eLife.107745.3.sa1

Summary:

In this manuscript, the authors reveal that the availability of extracellular asparagine (Asn) represents a metabolic vulnerability for the activation and differentiation of naive CD4+ T cells. To deplete extracellular Asn, they employed two orthogonal approaches: activating naive CD4+ T cells in either PEGylated asparaginase (PEG-AsnASE)-treated medium or custom-formulated RPMI medium specifically lacking Asn. Importantly, they demonstrate that Asn depletion not only impaired metabolic reprogramming associated with CD4+ T cell activation but also reduced CD4+ helper T cell lineage-specific cytokine production, thereby ameliorating the severity of experimental autoimmune encephalomyelitis.

The experiments presented here are comprehensive and well-designed, providing compelling evidence for the conclusions. The conclusions will be important to the field.

Comments on revised version:

The authors have sufficiently addressed my previous comments. The manuscript represents an excellent contribution to the field.

# Referee report: Reviewer #2 (Public review)

**DOI:** 10.7554/eLife.107745.3.sa2

While the importance of asparagine in the differentiation and activation of CD8 T cells has been previously reported, its role in CD4 T cells remained unclear. Using culture media containing specific amino acids, the authors demonstrated that extracellular asparagine promotes CD4 T cell proliferation. Consistent with this, depletion of extracellular asparagine using PEG-AsnASE suppressed CD4 T cell activation. Proteomic analysis focusing on asparagine content revealed that, during the early phase of T cell activation, most asparagine incorporated into proteins is derived from extracellular sources. The authors further confirmed the importance of extracellular asparagine in vivo, demonstrating improved EAE pathology.

While the data are well organized and convincing, the mechanism by which asparagine deficiency leads to altered T cell differentiation remains unclear. It is also necessary to investigate the transporters involved in asparagine uptake. In particular, elucidating whether different T cell subsets utilize the same or distinct transport mechanisms would provide important insight into the immunoregulatory role of asparagine.

Comments on revised version:

The authors have addressed the previous concerns, and the manuscript has been significantly improved.

# Author comment: Author response

**DOI:** 10.7554/eLife.107745.3.sa3

**Authors:** Peter Georgiev, Sheila Johnson, Kiran Kurmi, Song-Hua Hu, SeongJun Han, Dillon Patterson, Thao Nguyen, Linglin Huang, Dan Liang, Naomi Goldman, Thomas Conway, Hannah Creasey, Jared Rowe, Marcia C Haigis, Arlene H Sharpe

**Affiliations:** Harvard Medical School Boston United States; Harvard Medical School Boston United States; Harvard Medical School Boston United States; Harvard Medical School Boston United States; Harvard Medical School Boston United States; Harvard Medical School Boston United States; Harvard Medical School Boston United States; Harvard Medical School Boston United States; Harvard Medical School Boston United States; Harvard Medical School Boston United States; Harvard Medical School Boston United States; Harvard Medical School Boston United States; Harvard Medical School Boston United States; Harvard Medical School Boston United States; Harvard Medical School Boston United States

The following is the authors’ response to the original reviews.

**Public Reviews:**

**Reviewer #1 (Public review):**

Summary:

In this manuscript, the authors reveal that the availability of extracellular asparagine (Asn) represents a metabolic vulnerability for the activation and differentiation of naive CD4+ T cells. To deplete extracellular Asn, they employed two orthogonal approaches: activating naive CD4+ T cells in either PEGylated asparaginase (PEG-AsnASE)-treated medium or custom-formulated RPMI medium specifically lacking Asn. Importantly, they demonstrate that depletion not only impaired metabolic reprogramming associated with CD4+ T cell activation but also reduced CD4+ helper T cell lineage-specific cytokine production, thereby ameliorating the severity of experimental autoimmune encephalomyelitis.

Strengths:

The experiments presented here are comprehensive and well-designed, providing compelling evidence for the conclusions. The conclusions will be important to the field.

We thank the reviewer for their assessment of our work and enthusiasm towards our findings.

Weaknesses:

(1) EAE is the prototypic T cell-mediated autoimmune disease model, and both Th1 and Th17 cells are implicated in its pathogenesis. In contrast, Th2 and Treg cells and their associated cytokines (such as IL-4 and IL-10) have been shown to play a role in the resolution of EAE, and potentially in the modulation of disease progression. Thus, it will be important to determine whether Asn depletion affects the differentiation of naive CD4+ T cells into corresponding subsets under Th2 and Treg polarization conditions, as well as the expression of lineage-specific transcription factors and cytokine production.

We appreciate that the reviewer recognizes the functional relevance of our findings showing that Asn is important for proper Th17 differentiation and promotion of EAE (Figure 5 E-J, Figure 6). Given that multiple CD4+ T cell subsets play a role in both the initiation and resolution of EAE, we agree that it would be valuable to further support these findings with complementary Th2 and Treg differentiation experiments.

To address this, we examined the effects of asparagine depletion during in vitro iTreg and TH2 differentiation. We found that the frequencies of FOXP3+ iTreg and GATA3+ Th2 cells were reduced when cultures were grown in asparagine-deficient media. These results have been added to Supplementary Figure 5.

(2) EAE is characterized by inflammation and demyelination in the central nervous system (CNS), leading to neurological deficits. Myelin destruction is directly correlated with the severity of the disease. For Figure 6, did the authors perform spinal cord histological analysis by hematoxylin and eosin (H&E) or Luxol fast blue (LFB) staining? This is important to rigorously examine pathological EAE symptoms.

We agree with the reviewer that histopathology including H&E and/or LFB staining is a useful indicator of EAE disease severity. However, we are no longer able to obtain PEGAsnASE (Oncaspar) to perform these studies.

**Reviewer #2 (Public review):**

While the importance of asparagine in the differentiation and activation of CD8+ T cells has been previously reported, its role in CD4+ T cells remained unclear. Using culture media containing specific amino acids, the authors demonstrated that extracellular asparagine promotes CD4+ T cell proliferation. Consistent with this, depletion of extracellular asparagine using PEG-AsnASE suppressed CD4+ T cell activation. Proteomic analysis focusing on asparagine content revealed that, during the early phase of T cell activation, most asparagine incorporated into proteins is derived from extracellular sources. The authors further confirmed the importance of extracellular asparagine in vivo, demonstrating improved EAE pathology.

While the data are well organized and convincing, the mechanism by which asparagine deficiency leads to altered T cell differentiation remains unclear. It is also necessary to investigate the transporters involved in asparagine uptake. In particular, elucidating whether different T cell subsets utilize the same or distinct transport mechanisms would provide important insight into the immunoregulatory role of asparagine.

(1) The finding that asparagine supplementation promotes T cell proliferation under various amino acid conditions is highly significant. However, the concentration at which this effect occurs remains unclear. A titration analysis would be necessary to determine the dosedependency of asparagine.

Our studies indicate that the concentration of asparagine present in conventional RPMI lymphocyte media is sufficient to support CD4+ T cell activation and proliferation in vitro (Figure 1, Supplementary Figure 1 & Figure 2). This concentration was consistently used throughout our studies. In line with the reviewer’s comments, however, we have not yet determined the dose dependency of Asn during CD4+ T cell activation.

To address this, we performed a titration experiment in which asparagine was supplemented at varying concentrations in DMEM and Asn-deficient RPMI. Activation markers were measured 24 hours after TCR stimulation under these culture conditions. We found that the critical asparagine concentration lies between 37.8 and 3.78 uM. This concentration range is consistent with the physiological concentration of asparagine in murine plasma, which is approximately 50 uM (PMID: 24842860; PMID: 23853755). These data have been added to Supplementary Figure 1.

(2) The effects of asparagine deficiency occur during the early phase of T cell activation. Thus, it is likely that the transporters responsible for asparagine uptake are either rapidly induced upon activation or already expressed in the resting state. Since this is central to the focus of the manuscript, it is interesting to identify the transporter responsible for asparagine uptake during early T cell activation. A recent paper (DOI: 10.1126/sciadv.ads350) reported that macrophages utilize Slc6a14 to use extracellular asparagine. Is this also true for CD4+ T cells?

While a comprehensive characterization of the amino acid transporter network is certainly of interest, it is beyond the scope of the present study. As the reviewer notes, others have explored asparagine transport in lymphocytes. For example, Wu et al. (PMID: 33420490) determined that the asparagine transporter, Slc1a5, is significantly upregulated in CD8+ T cells upon activation, based on qRT-PCR measurements comparing mRNA from naïve and activated CD8+ T cell. They further validated the functional role of Asn transporters in CD8+ T cells by measuring N15-labeled asparagine uptake in the presence of siRNAs targeting the asparagine transporters Slc1a5 or Slc38a2 and found that inhibition of either transporter significantly reduced intracellular N15-Asn accumulation.

To gain additional insight into Asn transporters in distinct CD4+ T cell subsets, we reanalyzed a published RNA-seq dataset (Thakore et al., 2024; PMID: 39009838). We quantified the expression of transporters Slc1a5, Slc38a2, and Slc6a14 in naïve and activated CD4+ T cells polarized under Th1, npTh17, or pTh17 conditions at various time points. We observed that Slc1a5 expression increased upon activation in all subsets. Similarly, Slc38a2 expression increased during early activation stage, but subsequently returned to basal levels similar to naïve cells. In contrast, Slc6a14 showed relatively low basal expression in naïve cells compared to the other transporters investigated, and its expression decreased over the differentiation period in all CD4+ T cell subsets examined. These results indicate that Asn transporters Slc1a5 and Slc38a2 are expressed in CD4+ T cells during early activation and differentiation. These data have been included in Supplementary Figure 3.

(3) Given that depletion of extracellular asparagine impairs differentiation of Th1 and Th17 cells, it is possible that TCR signaling is compromised under these conditions. This point should be investigated by targeting downstream signaling molecules such as Lck, ZAP70, or mTOR. Also, does it affect the protein stability of master transcription factors such as Tbet and RORgt?

We agree with the reviewer that asparagine deprivation could impact several aspects of T cell function. In our study, we demonstrate that asparagine is crucial for CD4+ T cell protein synthesis and the expression of activation markers (Figure 1B-K, Figure 2K-L, and Figure 3AC). We also highlight its importance in promoting CD4+ T cell subset differentiation and lineage-defining cytokine production (Figure 5B-J). Other studies have reported a role for asparagine in early activation marker expression in CD8+ T cells and in enhancing LCK function (PMID: 33822775; PMID: 33420490). Given its proposed function as a promoter of LCK signaling function in CD8+ T cells, it will be important to determine if a similar mechanism operates during CD4+ T cell activation in future studies.

We appreciate the reviewer’s inquiry regarding the stability of critical transcription factors defining Th1 and Th17 subsets. We have examined the expression of the transcription factors RORγT and Tbet in Th17 and Th1 polarized cells and observed reduced expression in the absence of asparagine. We have included these findings in Supplementary Figure 5.

(4) Is extracellular asparagine also important for the differentiation of helper T cell subsets other than Th1 and Th17, such as Th2, Th9, and iTreg?

Please see our response to Reviewer 1 regarding iTreg and TH2. Investigation of Th9 cells is beyond the scope of the present study.

(5) Asparagine taken up from outside the cell has been shown to be used for de novo protein synthesis (Figure 3E), but are there any proteins that are particularly susceptible to asparagine deficiency? This can be verified by performing proteome analysis, and the effects on Th1/17 subset differentiation mentioned above should also be examined.

The investigation of specific proteins that exhibit asparagine dependency would indeed be interesting. Given our results showing that global protein synthesis is blunted with asparagine deprivation (Figure 3A-C), it would be particularly compelling to identify proteins with a specific requirement for asparagine. However, this level of analysis is beyond the scope of our study.

(6) While the importance of extracellular asparagine is emphasized, Asns expression is markedly induced during early T cell activation. Nevertheless, the majority of asparagine incorporated into proteins appears to be derived from extracellular sources. Does genetic deletion of Asns have any impact on early CD4+ T cell activation? The authors indicated that newly synthesized Asns have little impact on CD8+ T cells in the Discussion section, but is this also true for CD4+ T cells? This could be verified through experiments using CRISPR-mediated Asns gene targeting or pharmacological inhibition.

We appreciate the reviewer’s consideration of the contribution of endogenous asparagine to CD4 +T cell function. However, genetic perturbation of Asns is beyond the scope of our study, which is specifically focused on defining the requirements for extracellular asparagine and its role in CD4+ T cell activation.
